# Integrating climate-smart practices in forestry: insights from Europe and America

**DOI:** 10.3389/fpls.2025.1583294

**Published:** 2025-07-17

**Authors:** Hongtao Xie, Mengyuan Chang, G. Geoff Wang, Yu Tang, Songheng Jin

**Affiliations:** ^1^ Jiyang College, Zhejiang A&F University, Zhuji, China; ^2^ College of Forestry and Biotechnology, Zhejiang A&F University, Hangzhou, China; ^3^ Department of Forestry and Environmental Conservation, Clemson University, Clemson, SC, United States

**Keywords:** climate-smart forestry, management techniques, greenhouse gas emission, smart harvesting operations, forest bioeconomy

## Abstract

Global climate change poses a great obstacle to the sustainability of world forestry, and the trifecta of enhancing forest stock, minimizing greenhouse gas emissions, and attaining sustainable forest management is still challenging. Climate-smart forestry (CSF), however, offers promising solutions to these issues, with its core objective being to foster sustainable development through enhanced forest resilience, reduced greenhouse gas emissions, and boosted forest productivity and income. This emerging focus on CSF seeks to understand the mechanisms of interactions between forest ecosystems and climate change and eventually find locally acceptable solutions. This review delves into the developmental objectives of CSF, providing a new insight into the latest research advances and practical experience in CSF among eight Europe and American countries, including Brazil, USA, Czech, Finland, etc. Meanwhile, we identify the main challenges that CSF is facing currently, including the climate change uncertainty, disconnection among policy, science, and practice, and trade-offs between different CSF objectives. To address these challenges, we proposed five potential aspects for CSF development and sketched their main applications. Specifically, Technological innovation and digital applications are highly encouraged, including GIS and remote sensing, Internet of Things (IoT), and artificial intelligence technologies. Besides, Intelligent logging operations and wood processing, forest bioeconomy should also be considered to promote the CSF development. The results offer new perspectives and strategies for mitigating climate change via sustainable forestry management and protecting forest economies and communities in the context of accelerated global climate change.

## 1 Introduction of climate-smart forestry

Long-term observation of the climate system in recent decades have unequivocally demonstrated a warming trend, which is primarily attributed to the large amount release of greenhouse gas (GHG) released from human activities (Checa-Garcia et al., 2016). Over the past 30 years, global surface temperature has escalated by about 0.2 °C per decade (Wang et al., 2022). The elevated GHGs have led to multifaceted implications for forestry development, including the increase of pest and disease outbreaks, and forest fire risks, while the decrease of ecosystem services (Bhatti et al., 2024). These impacts pose threats to forest production, ecosystem health, and ultimately, the sustainable development of forestry. Besides, the disturbances to forests, including natural and human-induced, have resulted in forest biomass loss, contributing to approximately 20% of annual global GHG emissions as estimated by IPCC (Song et al., 2019). Among them, land use changes, deforestation, forest fires, soil respiration, and other forest exploitation activities are the primary sources of GHG emissions. The rapid expansion of the global economy is also a fundamental driver of these emissions and the trend is predicted to be continued in the upcoming decades (Liu et al., 2019). Consequently, mitigating GHG emissions poses a great challenge for sustainable forestry development.

**Figure 1:**
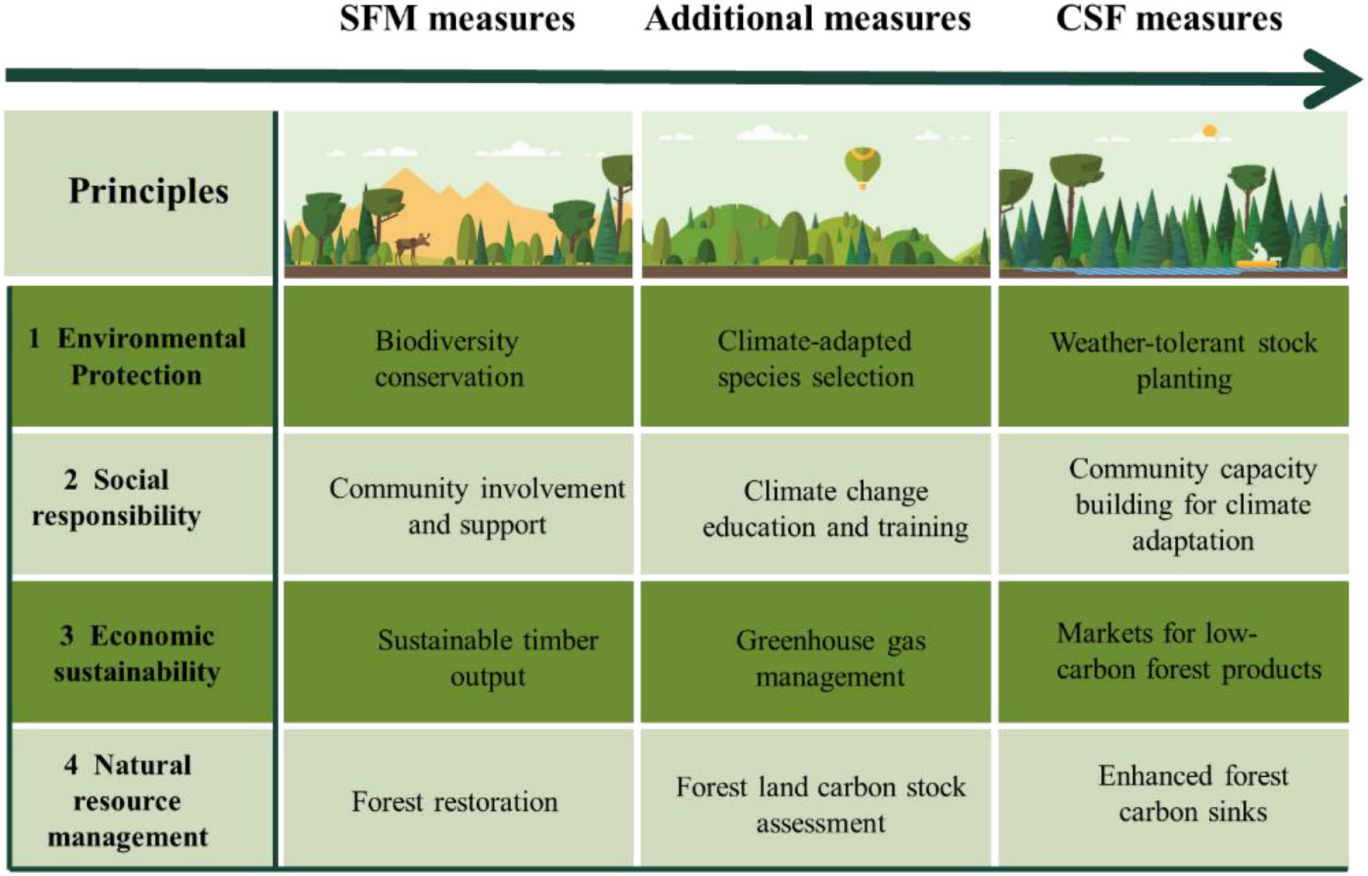
Framework of the developing goals from sustainable forest management (SFM) to climate-smart forestry (CSF).

Climate-smart forestry (CSF) was firstly appeared in peer-reviewed literature in 2017, and the concept was subsequently refined through engagements with various stakeholders (Weatherall et al., 2022; Wang et al., 2025). CSF is a set of comprehensive management strategies designed to address the challenges posed by climate change on forest ecosystems and resource management. These approaches integrate economic, social, and environmental considerations to alleviate the global threats of climate change, with its objective being to augment the resistance, recovery, adaptability, and productivity of these ecosystems while simultaneously mitigating the effects of climate change (Verkerk et al., 2020). CSF offers a holistic strategy that underscores the utilization of forests as a critical part in offsetting climate change within the framework of sustainable forest management (SFM), which is considered to safeguard and augment environmental values for both the present and future generations (**Figure 1**) (Siry et al., 2018). A fundamental challenge of SFM is the balance of multiple objectives simultaneously, resulting in inevitable trade-offs, but its core object has always been to guarantee the provision of diverse ecosystem services (Bowditch et al., 2022).

CSF has great potential to augment forest productivity, increase resilience and income, while reducing GHG emissions by fostering synergies of diverse forest uses and strengthening collaboration among stakeholders on global, national, and local levels (Nabuurs et al., 2017). The expansive scope of CSF encompasses multiple facets, including diverse tree species composition, expanded forest coverage, increased carbon storage, sustainable forest operation and management, and enhanced ecosystem services (Felipe-Lucia et al., 2018; Tognetti et al., 2022). More importantly, CSF incorporates the integration of modern forestry technologies, for instance, the implementation of multifunctional management and sustainable forest harvesting, has demonstrated their potential to increase productivity and decrease GHG emissions (Triviño et al., 2017; De Jong et al., 2017; Zhu et al., 2022). While the management of mixed-species plantations can enhance productivity by providing advantages in biodiversity, economics, and forest health (Liu et al., 2018; Mori et al., 2017).

The integration of cutting-edge technologies, such as remote sensing, the Internet of Things (IoT), and artificial intelligence (AI), has the potential to further elevate forest productivity. Remote sensing technology used in forestry can effectively monitor forest regeneration, fragmentation, changes of protected areas, genetic resource management, and provide insights to climate intelligence (Lechner et al., 2020). The synergistic combination of IoT and AI enables the detection of critical environmental parameters, such as weather, soil quality, and water conditions in forests (Garrido-Momparler and Peris, 2022). By utilizing AI algorithms for data analysis and prediction, real-time information regarding forest health, fire risk, and pest and disease warnings can be provided quickly, enabling prompt management and protective measures. For example, a comprehensive AI-IoT framework was introduced for monitoring the tree leaning in Hong Kong, offering an objective and efficient method to enhance the safety of urban forestry (Chau et al., 2023).

**Figure 2:**
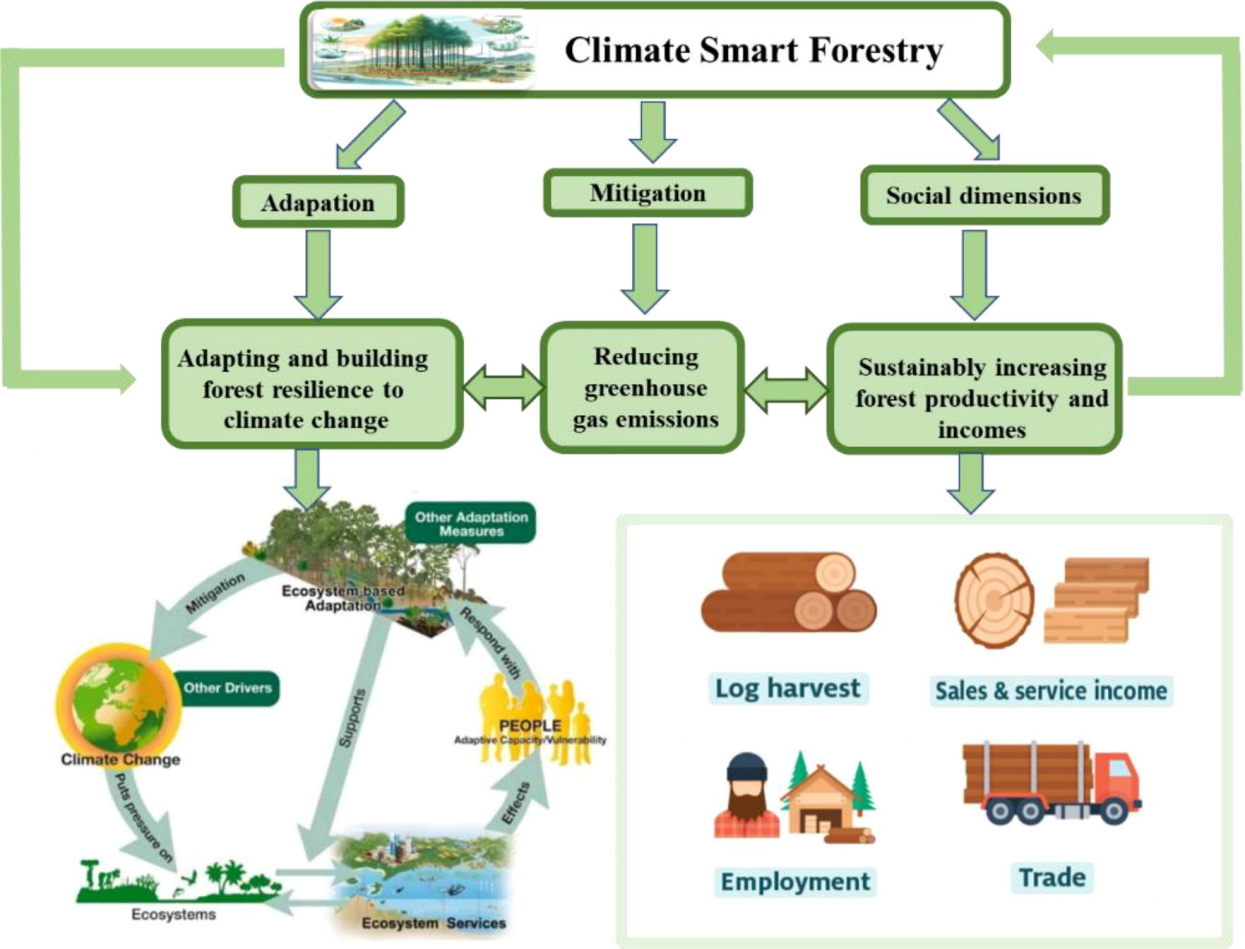
Main objects of climate-smart forestry.

**Figure 3:**
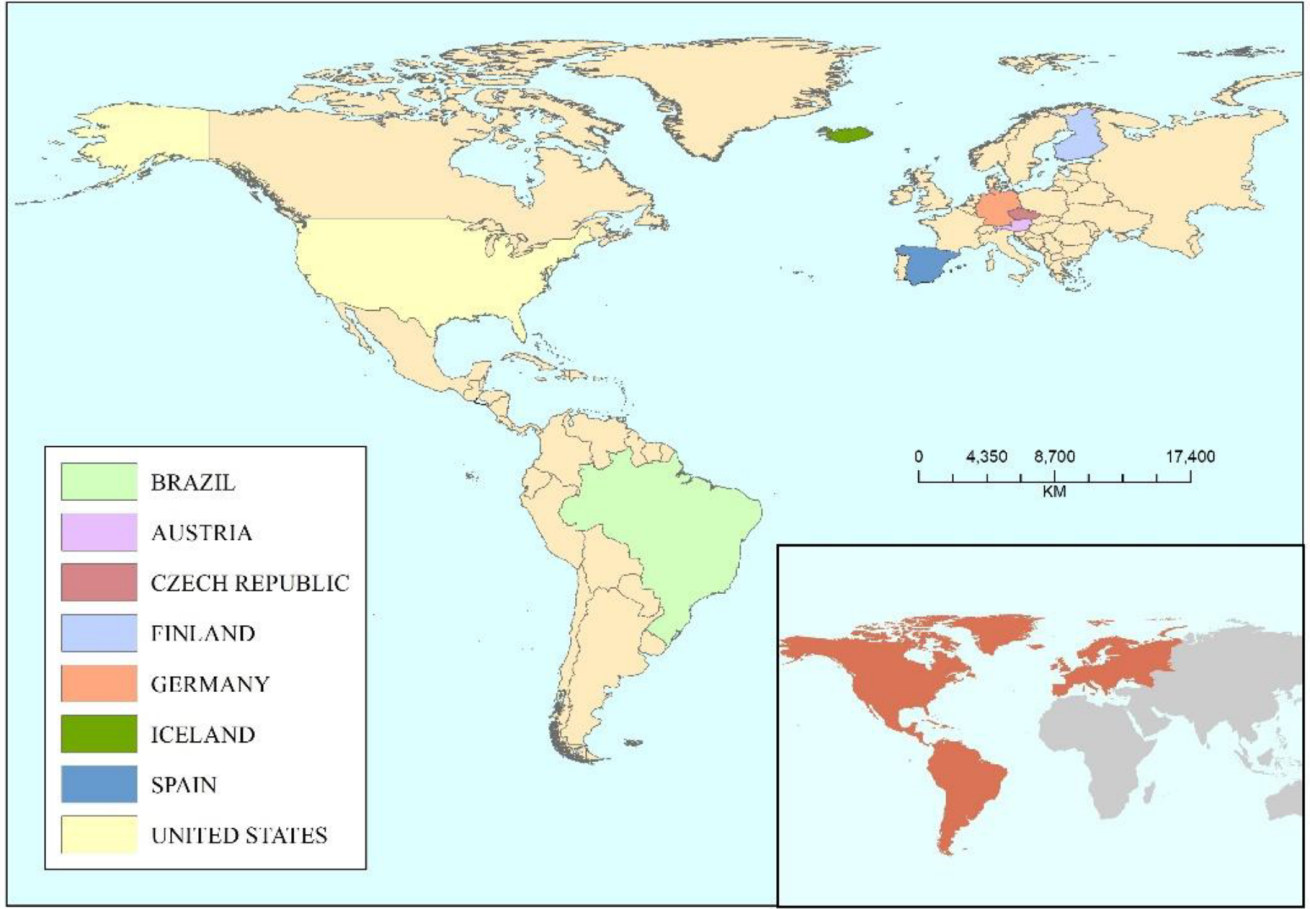
Countries selected for climate-smart forestry research in Europe and America.

There are three main objectives of CSF, including 1) reducing GHG emissions; 2) enhancing forest resilience to climate change; and 3) sustainably increasing forest productivity and incomes (**Figure 2**). The overarching aim of CSF is to manage forests in a climate-smart way by integrating considerations of climate change, ecosystem services, and socio-economic sustainability (Nabuurs et al., 2018). The ultimate goal of CSF is to achieve all the three objectives, but not all the measures implemented at each site will yield the desired outcomes (Yousefpour et al., 2018). Therefore, CSF must take a global perspective and consider all the three goals to find locally acceptable solutions (Pach et al., 2022). However, the importance of each objective will vary depending on the local context, requiring a balanced approach to prioritize the implementation of the goals of CSF (Jantke et al., 2016). This review outlined the developmental objectives of CSF, delving into the recent research advances in eight European and American countries (**Figure 3**, **Table 1**), while analyzing the challenges proposed. Finally, we proposed directions for the development of CSF, offering innovative ideas and strategies to address climate change and promote sustainable forestry management.

**Table 1:** Main climate-smart forestry practices in Europe and America.

Country	Forest cover (%)	Practical advances
Brazil	59	Integrated fire management; Financial incentive
USA	50	Forest product substitution; Incentives
Czech	34	Increasing wood utilization; Setting grants
Finland	86	Diversifying management strategies
Germany	33	Trade-off of forest profitability; Bioeconomy
Spain	55	Land-use modifications; Innovating forest assessment
Austria	20	Establish mixed forests; Selecting seed sources
Iceland	2	Incentive-based payment for ecosystem services

## 2 Recent advances of CSF in Europe and America

### 2.1 Brazil

Brazil is the second-largest forested country in the world, boasting an immense natural forest and plantation area spanning approximately 500 million hectares, which accounts for a staggering 59% of its total landmass (Giongo et al., 2022). Despite its natural bounty, Brazil ranks as the seventh-largest emitter of GHG globally, contributing 3.4% of the total emissions (Silva Junio et al., 2021). Land use changes and forestry activities accounted for 38% of total CO_2_ emissions in 2015 (Wu et al., 2015). The emission patterns in Brazil are greatly influenced by its deforestation trends, the driving forces include agricultural expansion, timber trade, population growth, road construction, and governance practices, with varying impacts across different regions (Silva Junio et al., 2021). Brazil now faces the challenge of balancing the preservation of ecosystem services with the demands of a growing population and community development.

To mitigate the impact of climate change, Brazil has implemented adaptation strategies to enhance forest resilience (Giongo et al., 2022). Among them, monitoring deforestation and forest fires through institutions like the National Institute for Space Research (INPE) has received great feedback. For instance, the collaboration between Brazil and China facilitated the development of CBERS program, which utilizes medium spatial resolution remote sensing imagery to monitor deforestation in the Amazon region (Picoli et al., 2020). These monitoring initiatives have yielded crucial data and insights that inform decision-making processes. Meanwhile, Brazil has conducted the National Forest Program (NFP) alongside the National Program for Rehabilitation of Native Vegetation (PLANAVEG), aiming to safeguard and restore the native forests, to obtain a balance between natural resource utilization and ecosystem preservation. Brazil is planning to restore at least 12 million hectares of native vegetation by 2030 and is taking proactive steps. Moreover, Brazil has established a financial incentive program for forest conservation, serving as a primary funding source for climate change mitigation and conservation strategies in tropical forest regions. Other forestry practices include ecosystem-based adaptation (EBA), and integrated fire management (Seroa da Motta et al., 2019).

### 2.2 United States of America

United States of America (USA) stands among the global leaders in terms of forested territories, with a total forest area spanning approximately 304 million hectares, nearly half of the country’s total landmass. The forest resources of USA are not only contributing significantly to the economy but also playing a crucial role in improving the environment. The CSF initiative’s primary objective can be effectively integrated into the existing forestry practices in USA. It is imperative to secure the policy support for diverse carbon storage paths, including deferred harvesting, reforestation, afforestation, pulpwood utilization, sawtimber utilization, and bioenergy utilization (Shephard and Maggard, 2023). Notably, policy backing for long-lived sawn timber products is particularly significant, as they offer the dual benefit of carbon sequestration and replacement of fossil fuel-intensive products (Shephard et al., 2022).

Forest product substitution has the potential to substantially enhance total carbon stocks by replacing fossil fuels and augmenting forest product inventories (Lippke et al., 2021). In the USA, southern fringe woodlands, for instance, may benefit from extended rotations due to their favorable forest productivity and robust regional timber markets. Conversely, Northeastern marginal lands, with lower forest productivity and timber markets, might be more suitable for reforestation aimed at producing woody biofuels (Stoof et al., 2015). Western forests, renowned for their high productivity but weaker timber markets, might find that forest lands with exceptional productivity are best suited for longer rotation cycles (Box and Fujiwara, 2021). Meanwhile, USA government has introduced incentives such as grants for climate-smart commodities in 2022. These initiatives aim to foster the expansion of forest product substitution in the USA (Page-Dumroese et al., 2022). Other CSF-related activities include large-scale tree planting, selective logging and thinning, controlled burns, and other fire management practices, as well as collaboration with international NGOs, universities, and the private sector to scale up CSF initiatives (McGann et al., 2023).

### 2.3 Czech Republic

Czech Republic boasts a forested area covering 26.7 million hectares, accounting for 34.1% of its total landmass (Hlásny et al., 2021a). These forests sequester 6.6 million tons of CO_2_ equivalent annually, thereby playing a crucial role in mitigating the impacts of climate change, but they are experiencing a decline in spruce-dominated stands, primarily due to the drought conditions and severe bark beetle infestation, posing a serious threat to both the forestry economy and environmental safety (Nabuurs et al., 2018; Bosela et al., 2021). Therefore, the top priorities for Czech are halting the decline of forests, reestablishing vegetation on deforested lands, and implementing adaptive management strategies, like increasing the proportion of broadleaf species, to develop resilient forest ecosystems that can better withstand the impacts of the changing climate and extreme weather events (Cienciala, 2022).

One of the key CSF practices in the Czech Republic is the conversion of unstable spruce (*Picea*) forests into more resilient forest types, such as beech (*Fagus*) and oak (*Quercus*), which fit the local conditions better and are more resistant to disturbances like droughts and windstorms (Cienciala and Melichar, 2024). This conversion reduces the area of spruce forests while enhancing the sustainability of harvests. Moreover, increasing the utilization of wood in long-term products helps to increase carbon storage (Schulze et al., 2020). Simultaneously, the utilization of spruce wood in various product categories is being differentiated based on whether it originates from stable or unstable forests to minimize carbon emissions from long-term products. The Czech government also supports CSF through policies and incentives, such as grants for forest restoration projects and programs to encourage private landowners to adopt sustainable forest management practices (Janová et al., 2022).

### 2.4 Finland

Finland is the most forested country in European Union, with 86% of its national territory classified as woodland. Over the last 50 years, the planting stock and increment have nearly doubled, largely due to the improvements in forest management practices (Peltola et al., 2022). However, these intensive management practices aiming at increasing timber production have produced negative impacts on forest biodiversity and the provision of ecosystem services. Besides, overuse of forest fertilization and maintenance of gully networks in peatland forests has also led to increased nutrient leaching and carbon emissions from the soil (Finér et al., 2021). The escalating threat of large-scale natural disturbances poses a risk of converting forests, at least in part, into carbon sources. Hence, Finnish forests necessitate various adaptation and risk management measures to bolster their resilience, including diversifying tree species, boosting forest regeneration and planting, refining water management, intensifying fire management and prevention, controlling pests and diseases, and fostering greater community engagement and awareness (Venäläinen et al., 2020; Peltola et al., 2022).

It is also imperative to consider the risk of wind damage when strategizing and executing thinning and meshing operations, avoid extensive thinning in high-risk zones, and take into account the risk of snow damage when applying fertilizers and conducting thinning activities (Laapas et al., 2019). Employing a set of management strategies, rather than a single approach, can enhance forest resilience (Díaz-Yáñez et al., 2020). Finland also mitigates the impacts of climate change by bolstering atmospheric carbon sinks within forests and leveraging them for wood production (Huuskonen et al., 2021). The magnitude of carbon sequestration in forests will be shaped by the level of forest management intensity and harvesting in response to wood demand in the forthcoming decades. Maximizing the climate benefits of harvested wood necessitates utilizing wood for products and fuels that yield lower GHG emissions (Hurmekoski et al., 2020; Gundersen et al., 2021).

### 2.5 Germany

Germany’s forests extend over 11.42 million hectares, constituting roughly a third of the nation’s entire landmass (Kleinn et al., 2020). These forests consist of a variety of types, including coniferous, broadleaf, and mixed forests. Renowned for their robust stocking and productivity, the German forests hold immense potential to resist climate change. Studies have shown their capacity to sequester GHG emissions is on par with or even surpasses the average level of Europe, making them the key players in the CSF (Hanewinkel et al., 2022). In recent years, Germany has lost substantial carbon due to droughts, bark beetles, and windstorms, all exacerbated by climate change. It becomes important to utilize the emissions-reducing capabilities of these forests while adapting to the changing climate.

Hanewinkel et al. (2022) highlighted the trade-off between forest profitability and *in situ* carbon sequestration, showing that using high-value, high-yield species like Norway spruce (*Picea abies*) for emission reduction comes at a high opportunity cost, while the adoption of low-value, high-yield species presents a more cost-effective approach. As a leading producer of sawn wood and wood panels, Germany stands poised to leverage these products to sequester carbon and replace energy-intensive materials, especially in the construction industry. It was estimated that the mitigation potential of wood substitution could be as significant as forest carbon sinks, potentially reducing up to 18 million tons of CO_2_ per year (Bösch et al., 2017). Recognizing the importance of bioeconomy, the German government has made strategic investments, including the establishment of a biorefinery in Leuna, which produces biochemicals for industries such as plastics, textiles, cosmetics, and industrial applications, serving as an alternative to fossil-based products (Gottinger et al., 2025). These initiatives contribute significantly to advancing the low-carbon transition and aligning with Germany’s CSF goals. Other measures for the forest sector to enhance their mitigation effect include the protection of forest soils, supervising small and medium private and community forest enterprises to reach climate protection goals etc.

### 2.6 Spain

Spain has 27.7 million hectares of forests, accounting for 55% of the country’s total land area currently (Vadell et al., 2016; Nabuurs et al., 2018). Broadleaf forests are the most predominant forest type, covering 56% of the total forested area, followed by coniferous forests at 37% and mixed forests at 7%, which spread across different climatic zones in Spain (Górriz-Mifsud et al., 2022). According to Land Use, Land-Use Change and Forestry (LULUCF), Spanish forests absorbed 11% of the country’s total GHG emissions in 2019 (Kazanavičiūtė and Dagiliūtė, 2023). However, drought and fire pose great threats to the forests in Spain and this trend is predicted to be continued, thus the mitigation strategies for fire should be implemented at various scales (Russo et al., 2017; Gil-Tena et al., 2019). These strategies include land-use modifications to create mosaics that could act as barriers against large fires, as well as preventative measures at the woodland level to slow down surface fires and prevent crown fires from occurring or spreading (Loepfe et al., 2012).

Many management options aimed at reducing competition for water resources (e.g. thinning) or enhancing water uptake efficiency (e.g. mixing species with preferred functional characteristics) can also help decrease fire risk (De Cáceres et al., 2021). In terms of forest resilience, Sánchez-Pinillos et al. (2016) introduced the Persistence Index (PI) to evaluate a forest’s ability to maintain functions and services post-disturbance. This index can guide the application of resistance and resilience concepts in practical forest management, supporting adaptive ecosystem management. Besides, the role of shrubs as nurse vegetation for pine (*Pinus*) seedlings has also been observed in semi-arid and arid Mediterranean and sub-Mediterranean pine forests, revealing its roles in reducing carbon emissions and enhancing sequestration (Sánchez-Pinillos et al., 2018).

### 2.7 Austria

Austria’s forested landscape accounted for about 20% of the country’s land area. These forests are primarily located in and around the Alps, while others are mainly distributed in the eastern hills and plains, including a variety of vegetation types such as coniferous, broadleaf, and mixed forests. Coniferous forests are more prevalent in the Alps, while spruce forests dominant in the lowlands and hilly areas, although their proportion has been decreasing since the 1980s (Lapin et al., 2019). The changing climate and poor forest management decisions, such as overstocking and over maturation, have resulted in a significant increase in bark beetles. Meanwhile, the rising temperatures have led to a 70 mm increase in evaporation rates in Austria over the past three decades, reducing the available water for plants and causing soil moisture deficiency. This makes the moderately drought-stressed trees more vulnerable to bark beetle attacks, particularly the northern Austrian spruce (Pasztor et al., 2014).

A feasible solution is to establish mixed-species forests to enhance tree diversity and reduce the risk of bark beetle and fire damage, transitioning from coniferous to broadleaf forests or adjusting crop rotation cycles, might enhance the forest stability (Hlásny et al., 2021b). Besides, selecting different seed sources or tree species also helps enrich the species diversity and promote the carbon sequestration potential. Australia’s sustainable forestry also tries to transform traditional timber production to bioenergy resources while maintaining vital ecosystem services. These innovations not only reduce waste and carbon emissions but also create new revenue for the regional communities (Ngugi et al., 2018). Smart harvesting employs satellite mapping and drone technology to identify optimal harvest areas and plan extraction routes with minimal environmental impact, which helps the foresters maintain crucial wildlife corridors and protect sensitive habitats (e.g., Tasmania’s Integrated Timber Energy Project).

### 2.8 Iceland

Iceland’s forest area only accounts for 1.9% of its land cover. These forests are predominantly located in the mountainous and river valley regions, particularly in the eastern and northern parts of Iceland, which are characterized by a lack of diversity, primarily consisting of coniferous and broad-leaved forests. This simplicity is attributed to Iceland’s harsh geographical and climatic conditions, which impose evident constraints on its forest development (Roy et al., 2018). In order to enhance the forest cover, mitigate GHG emissions, and foster economic development, Iceland has implemented a reforestation program. The afforestation initiative is designed to support the conservation of sustainable forests through the introduction of an incentive-based Payment for Ecosystem Services (PES) scheme (Brnkalakova et al., 2021).

The PES program incentivizes the conservation and sustainable management of forest resources by offering financial incentives to individuals who deliver ecosystem services, ensuring that forest management aligns with the CSF principles. Over the past 30 years, Iceland’s afforestation efforts have led to a 4.6% increase in its forested area, demonstrating the effectiveness of the program in expanding forest cover. Moreover, carbon stocks in Iceland’s forests and woodlands have surged by 40% in the last three decades, while net CO_2_ absorption has grown tenfold (Hunziker et al., 2019). Furthermore, Iceland’s afforestation program delivers economic benefits and other advantages to the local farmers (Olafsson et al., 2014). Through participation in the program, farmers receive complimentary seedlings, training sessions, and remuneration for their efforts. These incentives not only boost farmer engagement, but also generate positive externalities, which carry significant implications for climate change adaptation and mitigation.

## 3 Challenges for developing CSF

### 3.1 Uncertainty of climate change

Climate modelling has shown that the uncertainty associated with climate change is substantial and varies over time. It is therefore important to quantify the degree of time-varying uncertainty related to climate change at different levels, as this helps policymakers and investors in their decision-making (Moure et al., 2023). Climate change exerts both direct and indirect impacts on forests. The direct effects encompass modifications in forest growth conditions due to shifts in temperature, precipitation, and atmospheric CO_2_ levels, while the indirect impacts involve a range of abiotic and biotic disturbances (Venäläinen et al., 2020). Many studies have suggested that climate change’s effects on forests are ongoing and could be intensified in the future (Keenan, 2015; Mcdowell et al., 2016). Besides, the alterations in temperature and precipitation patterns result in changes in plant distribution, but the shaping effects would vary across regions, which amplifies the global effects of climate uncertainty (Seidl et al., 2017). With the increases in GHG emissions, many countries may face heightened vulnerability to extreme climatic events such as droughts, storms, pests, and diseases (Patel et al., 2024). Therefore, understanding how climate changes over time is crucial for the CSF development in the future (Fawzy et al., 2020).

### 3.2 Elevated anthropogenic GHG emissions

**Figure 4:**
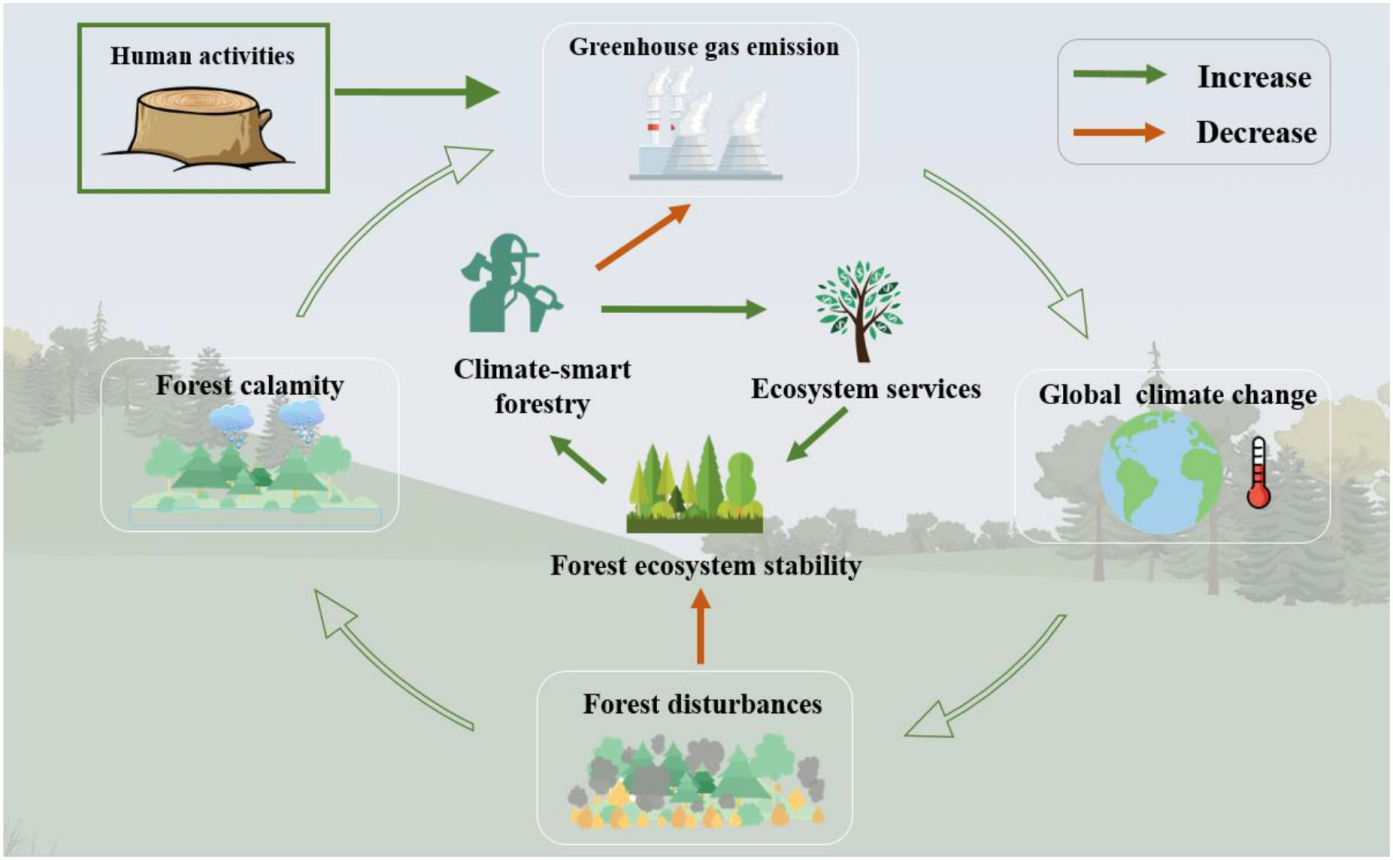
Role of climate-smart forestry in maintaining forest ecosystem stability and its relationship with global climate change.

Human activities and natural systems are major sources of GHGs, human source has been acknowledged through national commitments and GHG emission inventories, while the natural systems have higher levels of uncertainty (**Figure 4**). Land use changes, fossil fuel consumption, and forest degradation greatly contribute to the increase in anthropogenic GHG emissions (Arneth et al., 2017). For instance, forest degradation in developing countries, particularly in tropical and subtropical regions, is significantly contributing to the global GHG emissions (Requena Suarez et al., 2019). It has been estimated that forest degradation in 74 developing countries released 2.1 billion tons of CO_2_ annually between 2005 and 2010, with 53% attributed to timber harvesting, 30% to land use changes, and 17% to forest fires (Pearson et al., 2017). Although poorly managed forests could be a source of GHG emissions, most forests still hold significant potential for mitigating these emissions. Although various technologies have been used to control GHG emissions (clean energy alternatives, renewable energy technologies, etc.), enhance GHG uptake (e.g. carbon sequestration technologies; breeding new species) to adapt to the climate change, reducing anthropogenic GHG emissions is still a major challenge for developing CSF (Fan and Fang, 2023).

### 3.3 Disconnection between policy, science, and practice

An essential part of implementing the criteria and indicators for CSF is ensuring that forest managers can grasp the concepts and put them into practice (Weatherall et al., 2022; Bowditch et al., 2022). Nevertheless, navigating the intersection of policy, science, and practice has proven to be challenging, with many forest managers highlighting the significant communication gaps. While CSF definitions and indicators have been crafted by various forest professionals, the involvement of forest managers has been limited (Bowditch et al., 2020). Findings from an online survey revealed that 62% of forest managers found the CSF definition easy to comprehend, while 38% deemed it overly complex, suggesting that the original meaning may have been diluted during translation. Although forest managers welcomed CSF indicators, they expressed limitations in incorporating them into management plans due to insufficient knowledge and resources for measuring the full spectrum of indicators (Weatherall et al., 2022). It remains a considerable learning curve in extending CSF to the local level, which could involve further refining the concept through the integration of theory and practice and fostering knowledge exchange among individuals. It is possible to tackle climate change and other forest management challenges more effectively and facilitate the implementation of CSF by achieving a synergistic alignment of policy, science, and practice.

### 3.4 Synergies and trade-offs between different objectives

Forests are facing increasing social demands due to the changing environment and growing population. Forest managers must navigate multiple and sometimes conflicting objectives, such as providing forest products, conserving biodiversity, and sequestering carbon (Aggestam et al., 2020). A study conducted in European demonstrates that many forest management programs do not necessarily result in trade-offs between wood production, biodiversity, and carbon sequestration (Biber et al., 2020). Therefore, it is crucial to carefully consider the synergies and trade-offs between mitigation, adaptation, biodiversity conservation, and ecosystem services provision under uncertain climatic conditions and unpredictable extreme events (Tognetti et al., 2022). Customized CSF management strategies are essential to adapt to varying ecological and social conditions, as well as to evaluate interconnected impacts (Bowditch et al., 2020). These strategies should ensure the stability and sustainability of forest ecosystems, promote adaptation and mitigation efforts, and align with current objectives. Furthermore, they should be tailored to the specific conditions of different countries and regions, considering the unique circumstances present in each location.

### 3.5 Difficulties in data acquisition and monitoring system construction

CSF implementation necessitates substantial data for evaluating the forest’s adaptive capacity and vulnerability. The availability of reliable data and monitoring systems is crucial for assessing the effects of climate change on forests, thereby supporting the advancement of CSF practices (Temperli et al., 2022). Despite the implementation of numerous initiatives aimed at standardizing forest inventory estimates, challenges persist in the form of incomplete, inaccurate, or inconsistent data, thereby impeding the effective implementation and rigorous evaluation of CSF initiatives. For instance, the absence of standardized classification standards or guidelines resulted in varying classification methods by different data collection entities or individuals, leading to data classification discrepancies (Bowditch et al., 2020). This inconsistency complicates data comparison and integration, impacting data quality and reliability. Furthermore, forestry data often exhibit diverse formats and structures with a lack of standardization, posing challenges for data integration, analysis, and processing (Torresan et al., 2021). Addressing these hurdles is essential to enhance the assessment and management of the forest’s adaptive capacity and vulnerability to climate change impacts.

## 4 Implications for developing CSF

### 4.1 Technological innovation and digital applications

#### 4.1.1 Applications of remote sensing technology

Remote sensing technology has been widely utilized across various fields due to its obvious advantages, like real-time monitoring, extensive coverage, high resolution image etc. (Kerry et al., 2022; Chang et al., 2015). In the context of global change, remote sensing enables more comprehensive spatial and temporal monitoring of CSF by overcoming the difficulties of collecting field data in rugged landscapes and the seasonal constraints of accessing remote mountainous areas (Torresan et al., 2022). Remote sensing has emerged as a crucial tool for implementing CSF practices with a wide range of applications. For example, vegetation indices derived from optical satellite imagery have been utilized to estimate carbon stocks in aboveground biomass (Rodríguez-Veiga et al., 2017). Hyperspectral imagery has led to advancements in identifying vegetation types and their health state (Khan et al., 2018). In addition to infrared aerial photography, airborne hyperspectral systems and satellite observations have also shown valuable insights into the monitoring of forest alterations (Hill et al., 2019; Fraser and Congalton, 2021). For instance, Meiforth et al. (2020) employed spectral indices like NDVI and red-to-green ratios in combination with WorldView-2 and LiDAR to track kauri dieback in New Zealand. Among aerial mapping techniques, LiDAR or airborne laser scanning (ALS) has emerged as an efficient method for forest inventories (Goodbody et al., 2019). By applying algorithms to LiDAR data and employing adaptive robust filtering, researchers were able to enhance the effectiveness of land cover classification (Chen et al., 2023).

Although remotely sensed data have a wide range of applications in areas such as earth observation and environmental monitoring, there are still some drawbacks and limitations. The resolution of remotely sensed data is constrained by factors like sensor technology and satellite orbit altitude. Data is typically gathered by satellites or aircraft for periodic observation, leading to potential spatial and temporal sampling irregularities. The temporal resolution of remotely sensed data is tied to data collection frequency, with lower resolutions possibly missing short-term surface changes, limiting dynamic process monitoring and analysis (Abd El-Ghany et al., 2020). Furthermore, the high operational costs of satellites and aircraft, along with the need for specialized equipment and software for data acquisition and processing, elevated the overall cost and complexity of data collection. Therefore, the integration of multi-source remote sensing data could be a crucial direction for CSF development. By merging data from various sources with different spatial and temporal resolutions, it becomes feasible to procure and offer more precise and comprehensive surface and spatial information to bolster decision-making (Thapa et al., 2023).

#### 4.1.2 Using Internet of Things technology for data processing

**Figure 5:**
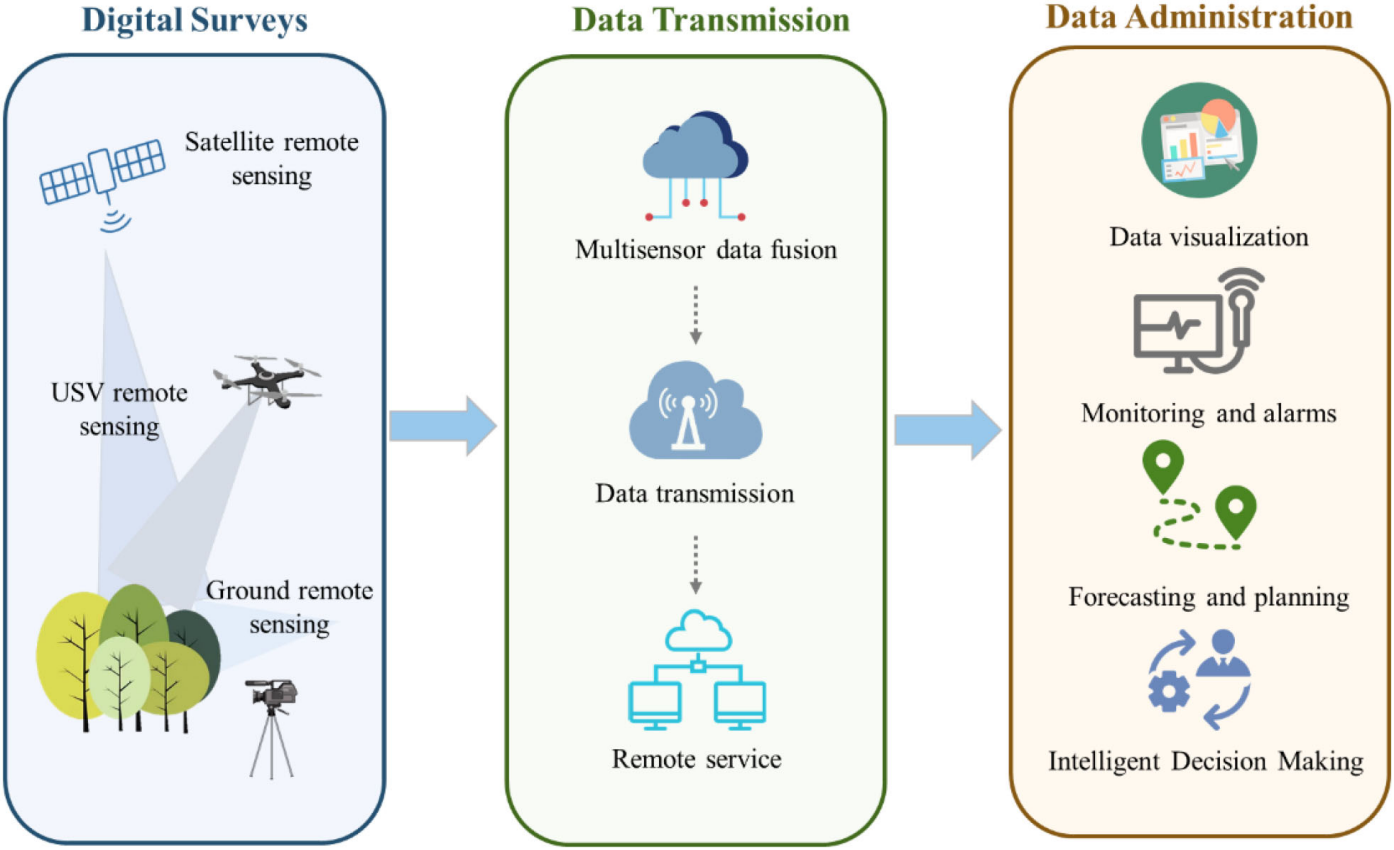
Role of multi-source remote sensing data in climate-smart forestry.

The emergence of Internet of Things (IoT) technology encapsulates a vast network of interconnected computing devices, sensors, and machines that are seamlessly intertwined with the Internet, each device within this intricate web boasts the remarkable capability to facilitate remote sensing and monitoring (Li et al., 2019; Ray, 2018). Regarding the forestry management, IoT technology primarily harnesses a diverse array of sensors to gather crucial data, transmitting data instantaneously to a centralized control unit through an integrated monitoring network for further process, enabling a comprehensive evaluation of the performance of trees and forests (**Figure 5**). Extensive research has been conducted on various facets of IoT integration in forestry, including multifunctional devices that leverage IoT systems, modular multifunctional devices, and the innovative fusion of decentralized structure of wireless sensor networks with the spatial precision of remotely sensed data within the forestry domain (Li et al., 2019; Zhao et al., 2022; Pause et al., 2016).

More IoT utilization in CSF is expected to prioritize more augmentation of security and privacy measures, which ensures not only the robustness of the system, but also the safety of users’ data. Advancing sensor technologies could be a focal point to achieve more precise and diverse data collection, IoT devices must facilitate unhindered data transmission and real-time sharing among devices (Wanyama et al., 2023). Besides, integrating big data processing and AI technologies further elevates the data processing efficiency and accuracy, e.g. by using data mining and machine learning, more insights can be extracted from vast datasets, thus augmenting decision-making. By giving precedence to these research and application, IoT can attain intelligent environmental monitoring, bolstering forestry production efficiency and sustainable development in CSF (Bradu et al., 2023).

#### 4.1.3 Using AI technology to manage forestry resources

Another promising trajectory for CSF lies in the integration of AI technology, the confluence of advancements in sensor technology, the pervasive availability of big data and cloud computing, the evolution of machine learning and deep learning algorithms, and the employment of GIS technology has offered new technical solutions for forestry resource management (Cheong et al., 2022; Hamano et al., 2023). AI is currently being implemented in many forestry domains, enabling the analysis of diverse forestry data and facilitating various functions, such as monitoring and managing forest resources, preventing and controlling pests and diseases, providing early warning and response to fires, and supporting ecosystem protection and restoration. AI can be harnessed to identify suitable geological formations for carbon storage, predict the behavior of CO_2_ within these sites, optimize injection procedures, and monitor storage sites to ensure the safe sequestration of CO_2_ underground. Consequently, AI contributes significantly to climate change mitigation by improving the prediction of extreme weather events, implementing sustainable forest management practices, and modeling nutrient cycling and plant productivity to reduce fertilizer use and minimize forestry risks (Zhao et al., 2023).

However, AI algorithms inherently demand vast, high-quality, and diverse datasets for their training and optimization processes. Forest managers are expected to use AI to enhance data collection and analysis capabilities, which helps assess risk factors such as fires, pests, diseases, and climate change, bolstering sustainable forest management by accurately predicting these factors. Besides, AI can spearhead the development of intelligent forestry practices and support the conservation and utilization of forest resources (Chen et al., 2020). Deep learning, a pivotal component of AI, elevates machine learning by increasing model complexity through harnessing deep learning technology, and forest managers can achieve intelligent monitoring, management, and protection of forest resources, enhance forestry production efficiency, and ultimately foster sustainable forestry development (Diez et al., 2021).

### 4.2 Improvement of agroforestry systems and planting techniques

Agroforestry systems that blend woody vegetation with plantations and livestock have great potential to bolster comprehensive production, food security, and mitigate the adverse effects of climate change (Fahad et al., 2022). The implementation of enrichment planting in agroforestry represents a land management strategy that not only elevates soil quality but also improves soil and water conservation. By introducing plant species that are indigenous to the local environment, the planting survival rate is significantly enhanced, thus contributing to the stability of the ecosystem. The application of livestock manure in forms such as pellets, compost, or biochar can significantly boost carbon sequestration and decrease GHG emissions (Sung-inthara et al., 2024). For instance, amending soil with biochar can enhance soil porosity, water retention, and nutrient availability, creating favorable conditions for plant growth (Hou et al., 2022). Increased biomass production facilitated by biochar can aid in carbon sequestration in vegetation and soil, while also conserving water, improving soil structure, and minimizing GHG emissions. It is important to acknowledge the variability in carbon sequestration and loss from soils, considering factors like soil type, vegetation, and climate in management practices to enhance carbon sequestration and minimize carbon loss (Huang et al., 2022). Therefore, the trajectory of agroforestry lies in fostering sustainable forestry development to achieve climate adaptation, GHG reduction, and the provision of ecosystem services. Refined planting techniques and optimized management practices are pivotal in realizing the comprehensive benefits of forestry.

### 4.3 Intelligent logging operations and wood processing

Currently, the development of CSF is steering towards sustainability, ecological balance, digitalization, and diversification. Advances in forest harvesting and wood processing with the integration of smart machinery are becoming increasingly paramount. Smart logging, a forefront innovation proposed by Hou et al. (2022) and Picchio et al. (2019), leverages sensors mounted on machinery to precisely evaluate tree attributes like shape, size, and weight. Technologies like load sensors, pressure sensors, stress wave propagation systems, near-infrared and hyperspectral imaging, as well as Radio Frequency Identification (RFID) tags and readers, empower the precise tracking and documentation of timber from logging to sawmill operations, these smart logging systems enhance not only timber quality assessment, but also minimize resource waste and environmental impact (Borz and Proto, 2022). Incorporating sensors, vision systems, and data analytics in wood processing enhances the measurement accuracy, product classification, and processing optimization, thereby augmenting the value and efficiency of wood utilization (Chang et al., 2015). The convergence of smart harvesting and wood processing operations, coupled with sensor data, offers invaluable insights for sustainable forest management. To obtain sustainable forest management goals, a robust tracking system from stump to sawmill, as well as the capability to manage vast amounts of data will be crucial in the future.

### 4.4 Incentive policies and measures

In the context of CSF, the implementation of incentives assumes a crucial role in catalyzing behavioral transformations (Brnkalakova et al., 2021). These incentives serve as a pivotal motivating factor for forest ecosystem service providers, encouraging them to adopt climate-smart management practices for the effective adaptation and mitigation of climate change (Gežík et al., 2022). A prevalent form of incentive is Payment for Ecosystem Services (PES), a voluntary transactional mechanism that ensures the delivery of specific ecosystem services by offering financial or non-financial rewards to the providers (Wegner, 2016). Within the realm of CSF, PES can incentivize forest practitioners to adopt climate-smart management strategies and measures. For instance, PES can provide financial support for activities like forest regeneration, enhancing biodiversity, and reducing carbon emissions. Other forms of incentives may include tax credits, subsidies, and reward programs, which help mitigate economic risks and offer financial benefits to practitioners. By combining both financial and non-financial incentives, this approach can foster heightened engagement and efforts among forest practitioners in CSF to reduce the impact of climate change (Grima et al., 2016). Therefore, the integration of incentives holds immense potential in propelling the advancement of CSF, and contributing significantly to the global effort against climate change, but more detailed plans are needed at the implementation level.

### 4.5 Developing forest bioeconomy

Forest bioeconomy is defined as the economic activities utilizing forest resources for sustainable development, which offer various opportunities and solutions for CSF (Letizia et al., 2023). Firstly, it incentivizes the use of sustainable wood and fiber resources, thereby minimizing the reliance on non-renewable resources. By creating high-value-added products from wood and fiber, the economic value of forest resources is enhanced while carbon emissions and environmental impacts are reduced (Jonsson et al., 2021). Secondly, forest bioeconomy promotes the development of bioenergy by utilizing forest biomass as a feedstock for biofuels and biogas, reducing GHG emissions and fostering sustainable energy development. Thirdly, forest bioeconomy contributes to biodiversity conservation and restoration through rational forest management, enhancing the resilience and adaptability of forests to climate change. Additionally, forest bioeconomy catalyzes green innovation and technology development (Giurca and Befort, 2023). By investing in research and the application of novel biomaterials, biochemicals, and biotechnology, the forest industry can be transformed and upgraded to adopt more sustainable and environmentally friendly production methods. Therefore, leveraging the economic, environmental, and social benefits of forest resources can lead to sustainable development amidst climate changes (Fischer et al., 2020; Giuntoli et al., 2022).

## 5 Conclusions

This study presents a comprehensive overview of the connotation, recent advances, challenges, and implications of CSF, a critical strategy to decelerate the impacts of climate change. CSF has achieved remarkable progress in Europe and America, not only in countries with rich forest resources like Brazil, the USA, Czech Republic, Finland, Germany, and Spain, but also in resource-limited nations such as Austria and Iceland. These advanced practices demonstrate diverse exploration paths and huge potential for CSF development. However, great challenges still exist in CSF practice, like the disconnect between policies, science, and practice, the synergies and trade-offs among different objectives, and the difficulties in establishing data acquisition and monitoring systems.

CSF holds immense potential in increasing forest stock and reducing GHG emissions, but we need to reinforce technological innovation and digital applications, optimize forestry management techniques to achieve intelligent timber processing, and formulate incentive policies to motivate ecosystem service providers to adopt climate-smart management practices. By integrating smart technologies and innovative management practices, CSF aims to ensure that forestry becomes more adaptive, resilient, and efficient in the face of climate change. By promoting the bioeconomy and intelligent harvesting, forest management can reduce waste, enhance productivity, and contribute to the circular economy. In conclusion, CSF represents a viable solution for mitigating the negative impacts of climate change on forestry. By addressing the current challenges and leveraging technological advancements, we can make use of the potential of CSF to foster a greener, more resilient, and sustainable ecosphere.
